# Domain-independent PPG signal quality assessment framework via representation learning

**DOI:** 10.1038/s41598-026-58783-6

**Published:** 2026-07-20

**Authors:** Rawan S. Abdulsadig, Esther Rodriguez-Villegas

**Affiliations:** Wearable Technologies Lab, Department of Electrical and Electronic Engineering, Imperial College London, London, SW7 2BT UK

**Keywords:** Photoplethysmography, Signal quality assessment, Representation learning, Transformers, Domain generalization, Computational biology and bioinformatics, Engineering, Health care, Mathematics and computing, Medical research

## Abstract

Photoplethysmography (PPG) is a widely used non-invasive sensing technique for monitoring cardiovascular and respiratory parameters, however, its measurements are highly susceptible to artifacts, which can degrade signal quality and, in turn, the accuracy of derived vital signs. Reliable PPG signal quality assessment (SQA) is therefore critical, yet existing SQA methods often rely on handcrafted features or ad-hoc thresholds and tend to be dataset-specific, limiting their generalizability across different sensor locations and users. To address these gaps, this paper proposes a novel SQA approach for PPG that is real-time compatible and sensor-location invariant. The method leverages deep representation learning by fine-tuning a pre-trained PPG transformer (PPG-PT) with a triplet loss to learn an embedding space where PPG segments are meaningfully partitioned based on their morphology. Signal quality is then quantified by computing each window’s embedding distance to a set of reference embeddings representing high-quality PPG segments, yielding a flexible reference-based quality score without requiring any model retraining when adapting to new devices or domains. The proposed framework was evaluated on three PPG datasets: the Complex System Laboratory (CSL) dataset providing finger PPG, the Wearable and Clinical Devices (WCS) for wrist PPG signals, and the Apnea and Artifacts (AA) dataset representing neck PPG signals, encompassing a wide range of artifact types and recording conditions. The model achieved strong agreement with expert annotations on the CSL dataset, with an F1-score of *≈*90%. It also demonstrated robust generalization on the WCS wearable dataset: using a generalized reference set drawn from multiple subjects, the approach attained a high F1-score of about *≈*95% on wrist PPG data, highlighting the method’s potential for population-level deployment. Performance on the AA neck PPG dataset was moderately acceptable with *≈*70% F1-score, likely due to the complexity and varying executions of the different artifacts presented in the dataset. The proposed methodology provides a deep learning SQA framework capable of generalizing across different PPG sensor locations and devices.

## Introduction

Photoplethysmogram (PPG) is a low-cost, non-invasive optical sensing technique widely used to monitor cardiovascular and respiratory activity. It enables the extraction of a variety of physiological parameters, including heart rate (HR), heart rate variability (HRV), oxygen saturation (SpO$_2$), respiratory rate, and even indicators of vascular health, making it a core component in both clinical monitoring and consumer wearable devices^1–3^.

Despite its utility, the reliability of PPG-derived measurements is highly dependent on signal quality, which can be significantly degraded by various artifacts such as motion, poor sensor contact, ambient light interference, and physiological factors like low perfusion. These artifacts can severely affect the accuracy of derived parameters and often go undetected in real-world applications. As such, the development of accurate, robust signal quality assessment (SQA) methods is a critical prerequisite for the safe and effective use of PPG in both clinical and wearable settings.

SQA for PPG remains a complex and non-trivial problem. The challenges span several dimensions: First, the morphology of a ‘clean’ PPG signal can vary substantially across sensor locations (e.g., finger, wrist, neck) and between individuals, complicating the creation of universal quality criteria. Second, many existing SQA methods rely on handcrafted features or thresholds, which often require dataset-specific tuning and may not generalize well to unseen users or devices. Third, the lack of standard benchmark datasets and consistent annotation practices further complicates both the development and validation of SQA algorithms.

A significant body of work focused on statistical or morphological signal quality indices (SQIs), sometimes coupled with classical machine-learning classifiers^4,5^. These methods offer interpretability and low computational overhead; however, their generalizability is often limited by the specificity of the chosen features and the investigated dataset. Although this makes way for promising avenues for deep learning frameworks to establish discriminative representations of PPG quality directly from raw signals, deep learning models are typically trained on isolated datasets or tailored to specific acquisition setups, making their generalization to different body locations or sensor types a continuing challenge. Furthermore, few existing methods are designed with real-time implementation in mind, and even fewer explicitly address the problem of generalizing quality assessment across body locations and use-case scenarios.

Domain adaptation has emerged as a prominent approach to address the cross-subject and cross-device variability inherent in PPG signals. These methods transfer knowledge from labelled source domains to a new target domain by aligning their feature distributions, typically through adversarial training or statistical discrepancy minimization, using unlabelled PPG data from the target subject to inform the adaptation^6^. While this removes the need for labelled target data, the model weights must still be updated using target-domain signals at deployment time, limiting practicality when rapid adaptation across heterogeneous devices or sensor locations is required. More broadly, recent advances in self-supervised representation learning for PPG signals have demonstrated that pre-training on large and diverse datasets can yield transferable embeddings that generalize across devices and recording conditions^7^. These foundation model approaches leverage morphology-aware contrastive objectives over millions of unlabelled segments to learn general-purpose representations, which are then adapted to specific downstream tasks via linear probing on labelled data. However, signal quality assessment has not been addressed as a downstream task in this context, and the general-purpose nature of the learned representations means they are not specifically optimized to discriminate between signal morphologies of varying quality.

To address these gaps, the main objective of this work is to develop a signal quality assessment methodology for PPG signals that is real-time compatible, sensor-location invariant, and does not rely on signal-specific handcrafted features. The developed approach in this study builds on the recently proposed PPG-PT model^8^, a pre-trained decoder-only transformer designed for PPG signals. The model is then fine-tuned using triplet loss metric learning as a task-specific objective that directly encodes morphological similarity and dissimilarity in order to learn embedding representations that reflect morphological distances between PPG windows. These embeddings are then used to assess quality based on their relative distances to a small set of reference embeddings assumed to correspond to high-quality segments. Unlike domain adaptation approaches that require model weight updates at deployment time, and unlike foundation model approaches that require labelled downstream data, the proposed method requires neither model retraining nor labelled data, adapting to new devices and sensor locations through a small reference set of high-quality signal segments that can be obtained, for instance, by asking the user to remain briefly stationary at the start of a monitoring session, or from a pre-acquired clean recording of the target device.

The proposed methodology in this work is validated across three datasets featuring signals from the finger, wrist, and neck, covering a wide range of acquisition conditions and artifact types. This work additionally presents both personalized and generalized reference configurations, allowing to assess the method’s adaptability and its potential for deployment in real-world wearable monitoring systems.

## Results and discussion

This section presents the experimental evaluation of the proposed reference-based signal quality assessment (SQA) framework across three datasets: the Complex System Laboratory (CSL), the Wearable and Clinical Devices (WCS), and the Apnea and Artifacts (AA) datasets, representing finger, wrist, and neck PPG recordings, respectively. The framework uses embeddings computed via The PPG-PT model fine-tuned with triplet loss. For all experiments, PPG signals were segmented into fixed-length windows and classified at the window level as either clean or artifact based on relative distances to a reference set in the learned embedding space. Performance was assessed using standard classification metrics (sensitivity, precision, F1-score, accuracy, and Cohen’s kappa) by comparing predicted labels against expert or protocol-based annotations. Unless otherwise stated, results are reported as mean ± standard deviation across subjects.

For the CSL and AA datasets, personalized reference sets were constructed from the initial windows of each recording, whereas for the WCS dataset, personalized references were drawn from finger PPG signals to emulate the use of pre-acquired reference data. In addition, a generalized reference configuration was evaluated, in which reference windows were pooled across multiple subjects to assess robustness and cross-subject generalization.

Across all datasets, a window was labeled as artifact (positive class) if *≥*10% of its duration overlapped with annotated artifact events, and as clean (negative class) otherwise.

### Evaluating the SQA methodology on the CSL dataset

Table 1 shows the resulting performance measures when evaluating the SQA methodology on the CSL dataset. The average measures over the two classes were found to be generally high, pointing to the method’s ability to successfully detect most of the artifacts in the signals of the two subjects. The SQA method achieved a mean sensitivity of *91.09± 0.32*%, mean precision of *88.99± 0.95*%, mean F1-score of *89.98± 0.39*%, overall accuracy and Cohen’s Kappa of *93.66± 0.38*% and *0.8± 0.01*, respectively.

**Table 1:** CSL dataset’s SQA performance measures for the negative class (clean), the positive class (artifact), as well as the average/overall measures

	Sensitivity %	Precision %	F1-score %	Accuracy %	Cohen’s Kappa
Negative (clean)	*95.23± 0.79*	*96.89± 0.28*	*96.05± 0.26*		
Positive (artifact)	*86.95± 1.42*	*81.1± 2.19*	*83.9± 0.51*		
Average/Overall	*91.09± 0.32*	*88.99± 0.95*	*89.98± 0.39*	*93.66± 0.38*	*0.8± 0.01*

While Table 2a and Table 2b show the individual confusion matrices of each of the two subjects ’Pox1’ and ’Pox2’, respectively, to give a closer view of the performance for each subject.

**Table 2:** Confusion matrices for subject ’Pox1’ (a) and subject ’Pox2’ (b) showing the detection results of the SQA methodology when applied to each.

(a) Subject ’Pox1’			
		Predicted	
		Positive (artifact)	Negative (clean)
**Actual**	Positive (artifact)	**599** **(87.96%)**	*82* *(12.04%)*
	Negative (clean)	*154* *(5.33%)*	**2739** **(94.67%)**

(b) Subject ’Pox2’			
		Predicted	
		Positive (artifact)	Negative (clean)
**Actual**	Positive (artifact)	**581** **(85.95%)**	*95* *(14.05%)*
	Negative (clean)	*122* *(4.21%)*	**2776** **(95.79%)**

It can be seen that the percentage of false negatives (FN) for both subjects were 12.04% and 14.05%, while the percentage of false positives (FP) were 5.33% and 4.21%, indicating that it was more capable in accurately detecting clean windows than artifacts (as observed in the average negative and positive sensitivities).

### Evaluating the SQA methodology on the AA dataset

Table 3 shows the evaluation results when applying the SQA methodology on the AA dataset, showing an overall accuracy of *71.96± 8.25*%, an average sensitivity of *71.81± 7.56*%, a precision *73.12± 7.84*%, F1-score *69.92± 8.04*%, and a Cohen’s kappa value of *0.42± 0.15*.

**Fig. 1:**
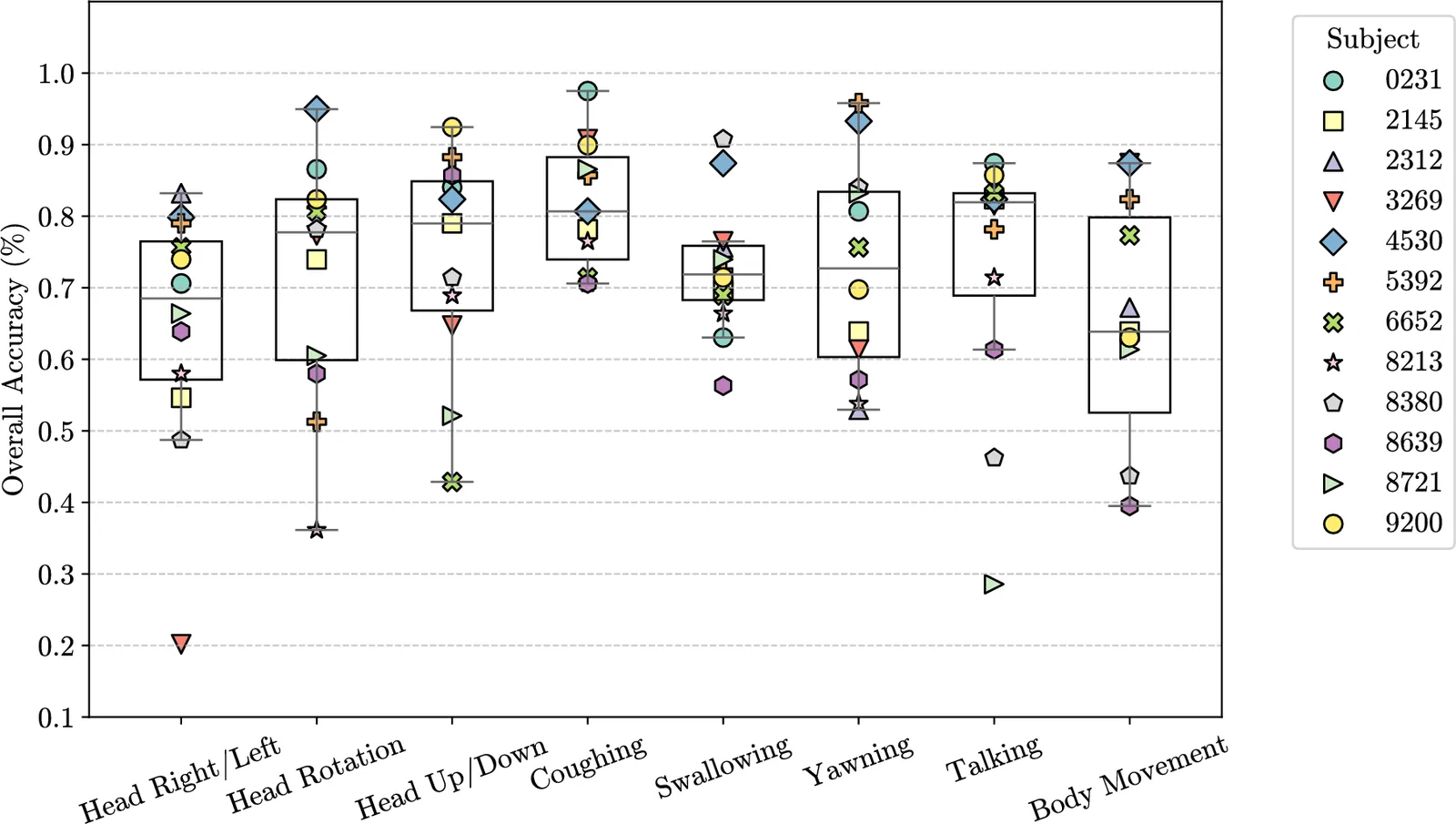
Boxplots showing the distribution of the overall accuracy when evaluating the SQA methodology on the AA dataset. The results are grouped by the different annotated artifact types, with the respective subjects uniquely marked as represented in the legend. Each box and whiskers shows the median (inner line), inter-quartile range (upper and lower ends of the box), and 10th and 90th percentiles (upper and lower whiskers).

Since the artifacts annotated in the AA dataset were divided into eight categories (coughing, swallowing, yawning, talking, head right/left, head rotation, head up/down, and body movements), this allows for the assessment of the robustness of the proposed methodology across varying artifact types and noise conditions. Figure 1 presents the subject-wise results per artifact type for the AA dataset.

**Table 3:** SQA methodology performance measures when evaluated on the AA Dataset, showing results for the negative (clean) and the positive (artifact) classes, as well as the average/overall measures

	Sensitivity %	Precision %	F1-score %	Accuracy %	Cohen’s Kappa
Negative (clean)	*74.74± 6.87*	*64.63± 10.29*	*66.88± 7.21*		
Positive (artifact)	*68.87± 12.81*	*82.2± 6.05*	*72.64± 10.21*		
Average/Overall	*71.81± 7.56*	*73.12± 7.84*	*69.92± 8.04*	*71.96± 8.25*	*0.42± 0.15*

It can be seen in Fig. 1 that the overall accuracy exhibited slightly varying per-subject distributions across the different artifact events, with a few notable outlier subjects in some of the events, such as subject 3269 in the head right/left event, and subjects 8721 and 8380 in the talking events. No clear systematic trends can be observed, however, it is notable that the distribution of the overall accuracy per subject in the coughing event was relatively high (*≈ 70*% - *≈ 98*%).

### Evaluating the SQA methodology on the WCS dataset

The following subsections present the results in the personalized and generalized schemes across the different configurations, when using the WCS dataset for evaluation.

#### Personalized reference set

Table 4 summarizes the results when sampling the reference set according to the personalized scheme, and when the number of samples was configured to be 10, 15, and 20 samples. A noticeable *≈ 2-4*% increase in the average sensitivity, specificity and F1-score as well as the overall accuracy, and a *≈ 0.1* in the Cohen’s kappa score was achieved when increasing the number of samples from 10 to 15. While increasing the number of samples from 15 to 20 did not improve the average/overall measures, however, it marginally decreased the variations of the measures across the subjects (represented by the standard deviations).

**Table 4:** SQA methodology performance measures when evaluated on the WCS Dataset via the personalized reference set scheme, across the three different reference sampling settings: 10, 15, and 20 samples. The table shows results for the negative (clean) and the positive (artifact) classes, as well as the average/overall measures, partitioned by configuration

	Sensitivity %	Precision %	F1-score %	Accuracy %	Cohen’s Kappa
**Reference set of 10 sampled windows**					
Negative (clean)	*93.22± 9.37*	*80.41± 24.52*	*83.56± 17.69*		
Positive (artifact)	*72.04± 40.7*	*92.34± 9.35*	*92.27± 8.92*		
Average/Overall	*82.63± 19.53*	*80.61± 24.0*	*83.95± 17.88*	*80.4± 23.91*	*0.65± 0.39*
**Reference set of 15 sampled windows**					
Negative (clean)	*92.98± 8.34*	*83.43± 22.42*	*85.78± 16.82*		
Positive (artifact)	*79.56± 32.15*	*92.31± 9.09*	*90.5± 11.4*		
Average/Overall	*86.27± 16.21*	*84.5± 21.48*	*85.74± 17.34*	*84.1± 21.53*	*0.72± 0.32*
**Reference set of 20 sampled windows**					
Negative (clean)	*92.41± 8.88*	*83.86± 22.48*	*85.62± 16.6*		
Positive (artifact)	*80.0± 32.37*	*92.26± 9.0*	*90.46± 10.86*		
Average/Overall	*86.21± 15.88*	*84.69± 21.23*	*85.65± 17.07*	*84.03± 21.3*	*0.72± 0.32*

**Fig. 2:**
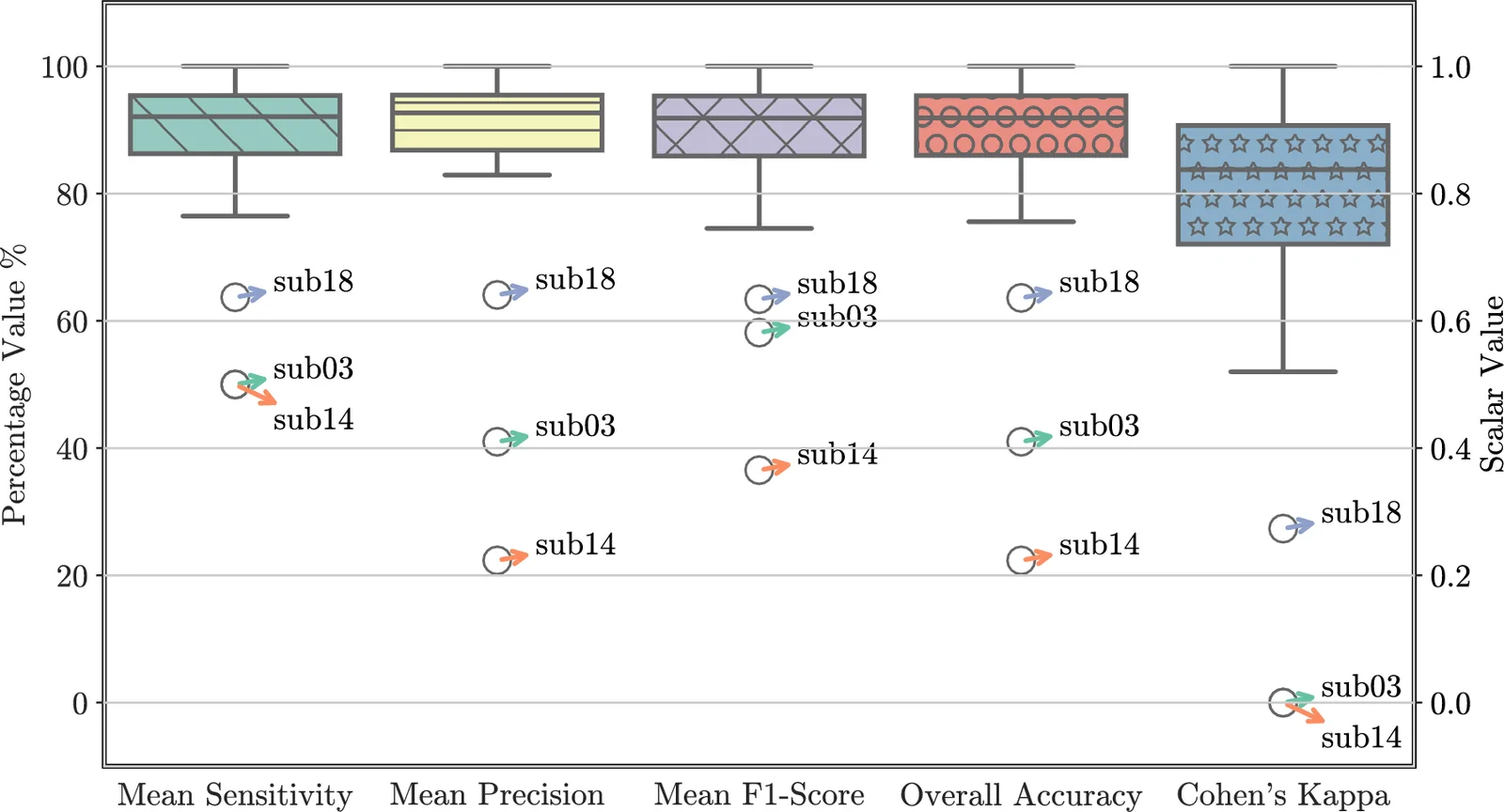
Boxplots showing the distribution of the detection metrics when evaluating the SQA methodology on the WCS dataset in the case of personalized reference sets of size 20 samples. The metrics shown are mean sensitivity, specificity, precision, F1, as well as the overall accuracy and Cohen’s Kappa metric, across the subjects, with the outlier subjects annotated. Each box and whiskers shows the median (inner line), inter-quartile range (upper and lower ends of the box), and 10th and 90th percentiles (upper and lower whiskers).

The distributions of the evaluation measures across the different subjects are shown in Fig. 2. It clearly shows that three subjects were driving the reduction of the evaluation results: sub18, sub03, and sub14. Particularly, sub03 and sub14 witnessed a sensitivity of 50% and a Cohen’s kappa score of 0.0, indicating that all windows were assigned the same class (‘artifact’ in these cases), which is most likely due to the great disparity between the PPG signals in their respective reference sets acquired from the stationary finger recordings, and the wrist evaluation recordings. This subject-level variability reflects the robustness limits of the personalized reference configuration under challenging cross-device conditions, where a large domain gap between the finger PPG reference signals and wrist PPG evaluation signals can drive all windows to be assigned the same class. The generalized reference configuration, presented in Section Generalized reference set, substantially mitigates this effect by pooling reference windows across multiple subjects.

#### Generalized reference set

**Fig. 3:**
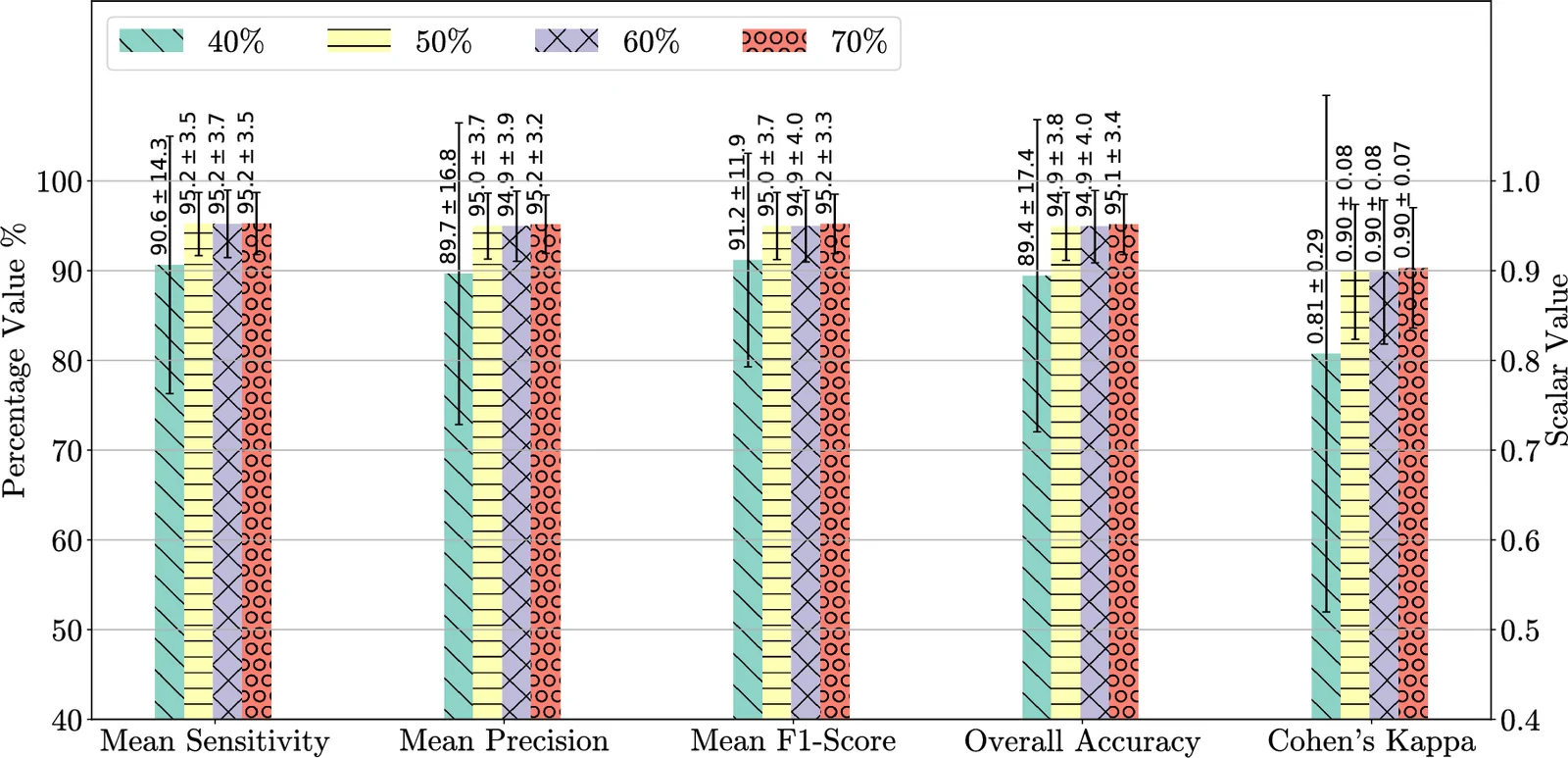
Barplots of the different evaluation metrics (mean sensitivity, mean precision, mean F1-score, overall accuracy, and Cohen’s kappa) when 5 reference windows were sampled from 40%, 50%, 60%, and 70% of the subjects.

As for the evaluation results corresponding to the WCS dataset when establishing a generalized reference set, the results associated with using 5 sampled reference windows per subject are shown in Fig. 3. The figure shows that the minimum results and maximum variance across all metrics were associated with using 40% of the subjects for the reference pool, with mean sensitivity equal to *90.6 ± 14.3*%, mean precision of *89.7 ± 16.8*, mean F1-score of *91.2 ± 11.9*%, overall accuracy of *89.4 ± 17.4*%, and Cohen’s kappa equal to *0.81 ± 0.29*. These results became significantly higher once the percentage of reference subjects was increased to 50%, adding a *≈ 5*% increase to the mean sensitivity, mean precision, mean F1-score, and overall accuracy, and a *≈ 0.1* increase in the Cohen’s kappa value. The figure also shows a notably insignificant difference between 50% and the higher percentages (60% and 70%). The considerable difference between the results when using 40% of the subjects compared to using 50%, 60%, or 70% can be due to the random selection of subjects and the heightened influence of sub-optimal reference windows when the size of the reference set was smaller compared to the increased reference set, coupled with the possibility of introducing more appropriate reference windows with the addition of new random reference subjects, which might have helped boost the generalizability of the methodology. Another likely possibility is that the method needed a representative collection of reference subjects, which (in this work) was at least equal to the number of target subjects in which the SQA methodology was being deployed.

These results were closely replicated when sampling 2 and 10 windows per reference subject, and are shown in the supplementary Figures S1 and S2, respectively.

**Fig. 4:**
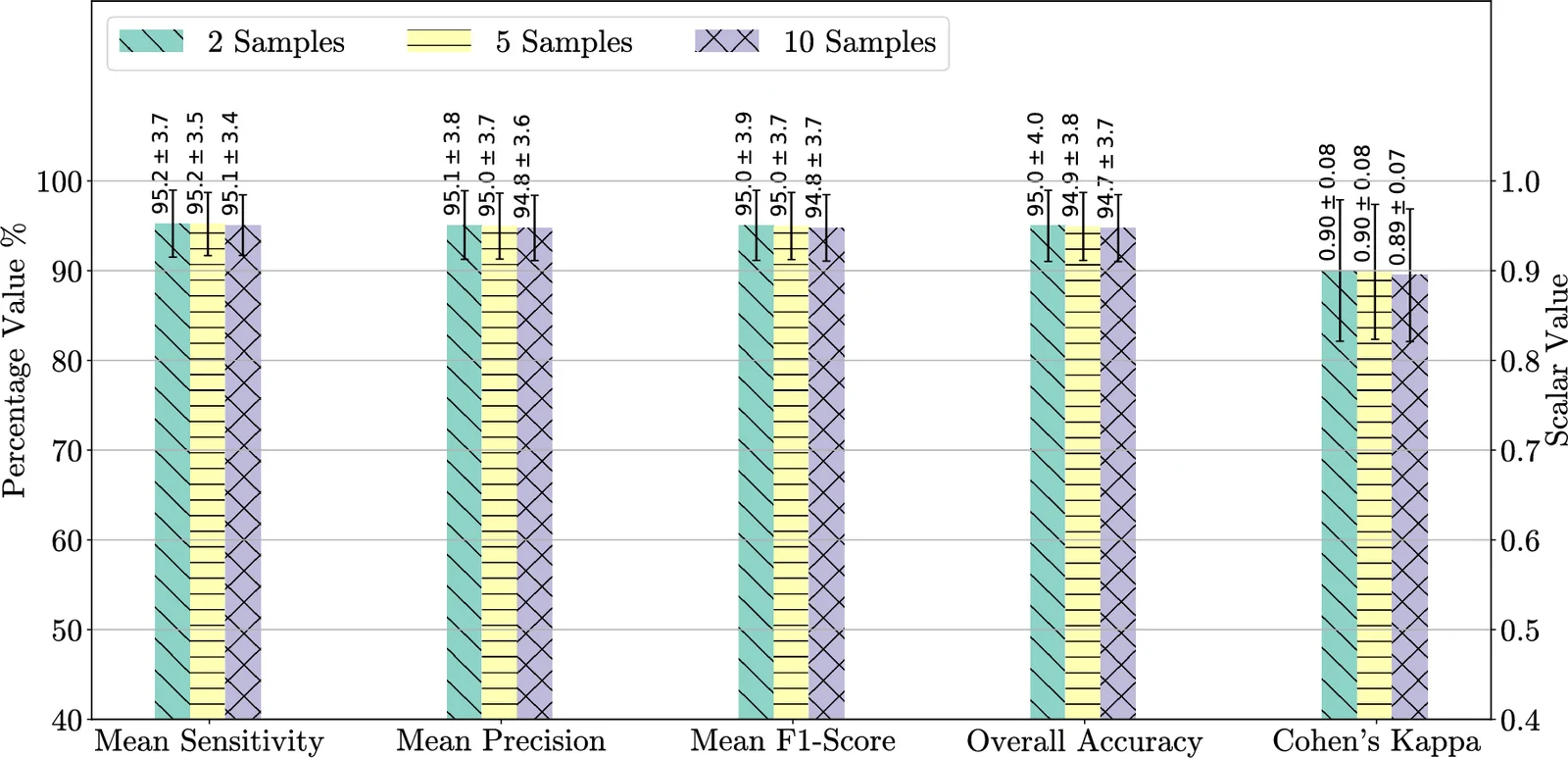
Barplots of the different evaluation metrics (mean sensitivity, mean precision, mean F1-score, overall accuracy, and Cohen’s kappa) when 50% of the subjects were randomly sampled, and 2, 5, or 10 samples were used in the reference set.

Figure 4 shows the evaluation metrics when 2, 5, and 10 random windows were sampled from 50% of the subjects. Interestingly, it can be seen that there was no notable difference in the performance across the three cases, with the choice of 2 samples from each subject being marginally higher than the 5 and 10 samples, with mean sensitivity, mean precision, and mean F1-score equal to *95.2 ± 3.7*%, *95.1 ± 3.8*%, and *95.0 ± 3.9*%, an overall accuracy of *95.0 ± 4.0*%, and a Cohen’s kappa value of *0.90 ± 0.08*.

Overall, when the SQA methodology was applied to the WCS dataset, the results showed a remarkable improvement in the performance when using a representative generalized reference set across subjects compared to a personalized reference set for each subject.

### Comparison with relevant published work

This section presents a comparison with previous published work that reported artifact detection results for any of the datasets used in this work. Although the ground truth assignment approaches may differ, the reported results can still give an idea of how they roughly compare to this work. Tables 5, 6 and 7 represent the complete set of published methods reporting artifact classification results on the CSL, AA and WCS datasets, respectively, to date, to the best of the authors’ knowledge. Table 8 then provides a structured comparison of the evaluation protocols across all methods, as differences in class definitions and evaluation schemes affect the interpretation of the reported performance.

**Table 5:** Comparable artifact detection results using the **CSL** dataset.

Method	Granularity	Annotations Threshold	Sensitivity (Pos)	Sensitivity (Neg)	F1-Score (Pos)	Overall Accuracy
Karlen et al.^9^	Pulse-level	-	41.1%	99.1%	52.1%	96.3%
Almeida et al.^10^	10 s window	10%	79.5%	97.1%	82.7%	93.9%
This Work (CSL)	10 s window	10%	86.9%	95.2%	83.9%	93.7%

Table 5 compares the CSL dataset results of the proposed methodology with relevant results presented in Karlen et al.^9^ and Almeida et al.^10^. Karlen et al.^9^ focused on classifying individual PPG pulses as ‘clean’ or ‘artifact’. Their algorithm, named Incremental-Merge Segmentation (IMS), segments the PPG signal into individual pulse beats and then classifies each beat as either a valid pulse or an artifact using adaptive morphological thresholds. On the other hand, Almeida et al.^10^ processed PPG segments in the form of 10 s windows, developed a signal qualifier algorithm based on the use of an adaptive threshold to identify Inverted Triangular Area (ITA) variations across the pulses, and classified the PPG window segments to ‘clean’ and ‘noisy’. The table shows that, although the specificity (sensitivity of the negative class) and the overall accuracy of the proposed method were less than the scores reported in the comparable papers (by up to *≈ 4*%), the sensitivity and F1-score of detecting artifacts were significantly higher, denoting the proposed method’s capabilities.

**Table 6:** Comparable artifact detection results using the **AA** dataset.

Method	Granularity	Annotations Threshold	Sensitivity (Pos)	Sensitivity (Neg)	F1-Score (Pos)	Overall Accuracy
García-López et al.^11^	6 s window	20%	85.43%	86.26%	85.80%	85.84%
This Work (AA)	10 s window	10%	68.9%	74.7%	72.6%	71.9%

As for Table 6, the AA dataset results presented in this work are compared against the results reported in García-López et al.^11^. In their work, a machine-learning approach using hand-crafted features and a support vector machine (SVM) classifier with RBF kernel was used to distinguish artifact-corrupted vs. clean PPG window segments.

The results shown could point to sub-optimal performance by the proposed methodology; however, given the machine-learning context, it is important to note that in their work, the positive class consisted of all artifact types grouped in one, while the negative class consisted of grouping apnea, normal, and slow breathing events, across the different subjects. While in this work, the positive class represented the artifact events, while the negative class consisted of the intermediate sections of the recordings between the artifact events, per recording.

Overall, the results of the proposed SQA methodology can be considered acceptable concerning the AA dataset, when the annotation and evaluation differences between the two studies are taken into account.

**Table 7:** Comparable artifact detection results using the **WCS** dataset.

Method	Granularity	Annotations Threshold	Sensitivity (Pos)	Sensitivity (Neg)	F1-Score (Pos)	Overall Accuracy
Jun et al^12^.	3 s window	– (visual)	89.8%	–	91.4%	92.6%
This work (WCS)$^\ddagger$	10 s window	10%	94.6%	95.9%	95.4%	95.0%

$^\ddagger$ Results obtained for the generalized reference set configuration using 2 samples from 50% of subjects.

Table 7compares the proposed methodology against Jun et al^12^. on the WCS dataset. The proposed method achieves an F1-score of *≈*95% under the generalized reference configuration, comparing favourably with the 91.4% reported by Jun et al. An important methodological distinction should be noted: Jun et al. employ leave-one-subject-out cross-validation, in which the LightGBM model is evaluated on 1 subject at a time and trained on the remaining subjects for each fold, effectively resulting in a different model for each fold due to the different training samples used. In contrast, the proposed method uses a frozen pre-trained model with a small reference set of assumed-clean windows randomly selected from out of sample subjects, requiring no retraining when adapting to a new device or dataset. These differences in evaluation paradigms are laid out in Table 8.

**Table 8:** Comparison of evaluation protocols across all methods and datasets.

Method	Dataset	Positive class	Negative class	Evaluation scheme
Karlen et al^9^.	CSL	Artifact beat	Valid beat	Rule-based; no training
Almeida et al^10^.		Noisy window	Clean window	Rule-based; no training
This work		Artifact window	Clean window	Frozen model; no retraining
García-López et al^11^.	AA	Artifact window (all types)	Normal + slow breathing + apnea$^\S$	Leave-30%-subjects-out; trained SVM; evaluated on remaining subjects
This work		Artifact window	Inter-event rest sections	Frozen model$^\ddagger$; no retraining
Jun et al^12^.$^\P$	WCS	“POOR” morphology	“GOOD” morphology	LOSO-CV; LightGBM trained and evaluated per fold
This work		Significantly distorted or noisy windows	Clean windows	Frozen model; no retraining

$^\S$The negative class in García-López et al. groups normal breathing, slow breathing, and apnea events together as clean PPG, whereas in this work the negative class consists of inter-event rest sections only.

$^\ddagger$The model was fine-tuned using apnea and sensor-rubbing recordings from the AA dataset; these recordings were excluded from evaluation. The frozen model was then applied directly to the remaining artifact recordings without further training.

$^\P$Results reflect the average of 18 separately trained models, one per left-out subject; no single model generalises across the full dataset without retraining.

Table 8 provides a structured overview of the key protocol differences across all compared methods, covering class definitions and evaluation schemes. The most notable differences are: (i) the class definition disparity between García-López et al. and this work on the AA dataset, as discussed above; and (ii) the fundamental difference in evaluation paradigm between Jun et al. and this work on the WCS dataset, where the former requires supervised training per deployment context and the latter does not.

## Methods

### Fine-tuning the base embedding model

This section describes the fine-tuning process carried out on the pre-trained decoder-only transformer model used in this work. The objective of this step is to adapt the model to produce embedding vectors that capture morphological differences between PPG signal segments in a manner that reflects signal quality. To achieve this, a triplet loss function is employed to guide the model in learning an embedding space where similar PPG windows (based on their morphology) are mapped closer together, while dissimilar ones are pushed further apart. The following subsections provide details on the base model architecture and the modifications introduced to enable this fine-tuning process.

#### The base model

The open-sourced generative pre-trained transformer (GPT) model PPG-PT was used as a base model. PPG-PT is a pre-trained decoder-only transformer model for PPG signals, consisting of 8 transformer blocks, each with 8 attention heads, in addition to a final Layer Normalization layer followed by a fully-connected Linear layer, resulting in an architecture of 443,943 trainable parameters^8^. This model was pre-trained on three datasets: The Capnobase dataset^13^, the BIDMC dataset^14^, and the cuffless blood pressure dataset^15^. The PPG-PT model’s generative capability was evaluated, and it was illustrated in their paper that the model is able to embed knowledge about PPG morphology, allowing it to extrapolate the PPG signal, given a PPG window, with somewhat similar properties.

#### Fine-tuning and model embeddings overview

In machine learning, it is often desirable to learn data representations that capture its essential characteristics in a lower-dimensional space. In a nutshell, embeddings are vectors that are able to provide these representations once the model is trained sufficiently. An embedding vector is the result of a function $f:X\rightarrow \mathbb {R}^{d}$ that maps the input data *X* into a d-dimensional continuous vector space, where *f* is the embedding model. A good embedding model would result in an embedding space that maintains useful spatial relationships between data points.

Fine-tuning is a popular technique in deep learning that allows pre-trained models to adapt to a different data distribution or problem. In the context of this work, fine-tuning is done to train the model, given its prior knowledge about PPG morphology, to produce embedding vectors that exhibit a clustering effect based on the characteristics of the PPG. In other words, two PPG segments would have embeddings that are close to each other if they show very similar morphological qualities, and would have embeddings that are far away from each other if they are very different. With that in mind, fine-tuning the model using triplet loss makes it possible to further achieve this objective.

Triplet loss is a type of loss function used for training neural networks, particularly in tasks involving metric learning. The goal of triplet loss is to push the embedded vector representations of similar data points closer together and to pull representations of dissimilar data points far away from each other. This method operates using a triplet of data points at a time, namely: anchor data point (A), positive data point (P), and negative data point (N). P and N refer to data points that are similar and dissimilar to A, respectively^16^.

**Fig. 5:**
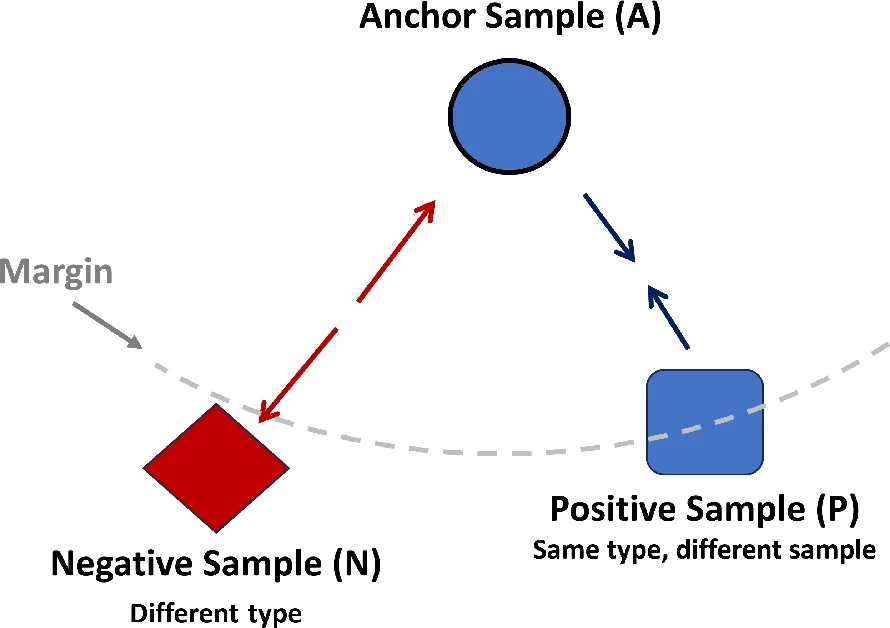
Illustrative figure of triplet loss operating on the triplet of data points A, P and N.

Fig.5 illustrates the idea of the triplet loss given the three data points A, P and N, as well as a margin. This loss trains the model in such a way that: $d(f(A) - f(P)) + \textit{margin} < d(f(A) - f(N))$, where *d* is a distance measure such as Euclidean distance, and *f* is the embedding model. The margin used in this work is 2. Furthermore, in cases where the data points P and N are mapped to embeddings that are closer to each other than P and A, a swap between data points A and P is performed where point P becomes the anchor and point A becomes the positive sample, then the loss is updated using the new assignments.

**Fig. 6:**
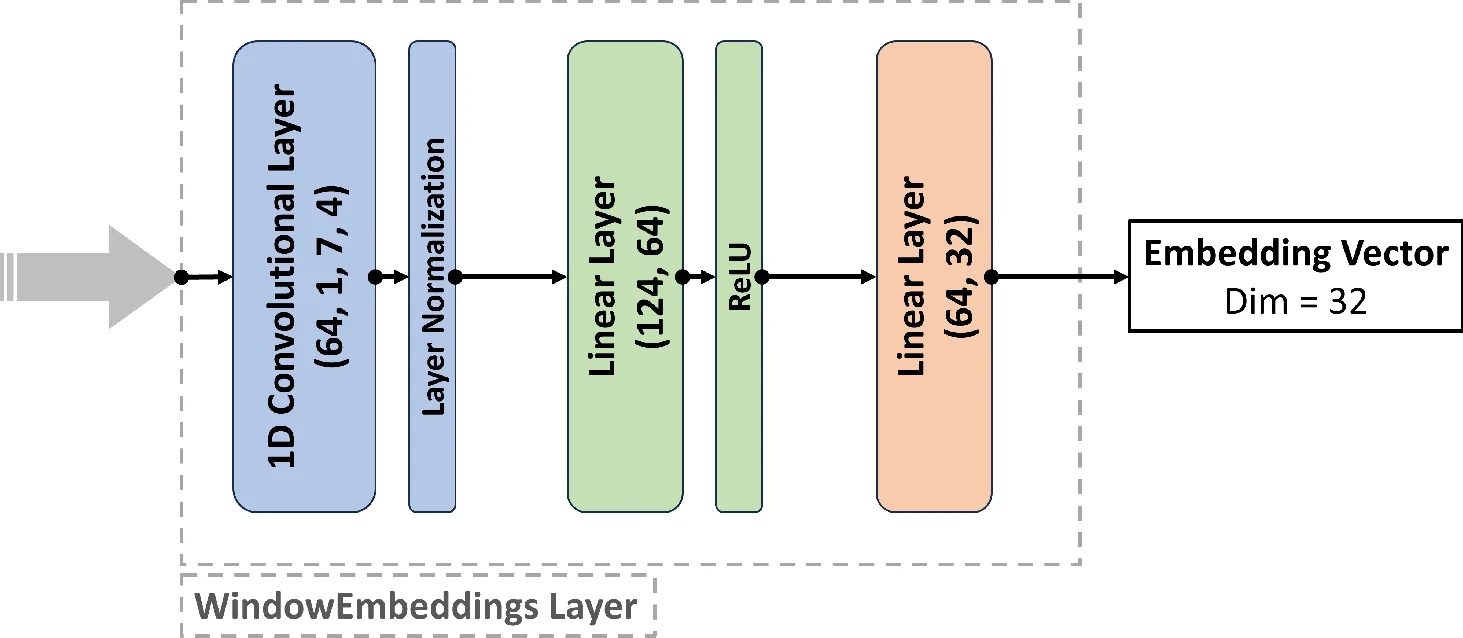
Descriptive figure of the WordEmbeddings layer, which produces the model’s output embedding vectors, consisting of a 1D convolutional layer (64 input channels, 1 output channel, kernel size of 7 and a stride of 4) followed by a layer normalization operation, a linear layer (input size of 124 and output size of 64) succeeded by a ReLU operation, and a final linear layer (input size of 64 and output size of 32).

Fine-tuning the base model for this work consisted of replacing the last linear layer with a new WindowEmbeddings layer depicted in Fig.6, containing a 1D convolutional layer followed by a layer normalization operation, a linear layer with a ReLU non-linearity operation, and a final linear layer that produces the output embedding vector. Following the authors’ recommendation for base model fine-tuning^8^; only the last transformer block, in addition to the final layer normalization and WindowEmbeddings layer, are trained, while the rest of the model weights are kept fixed.

#### Fine-tuning implementation details

All model weights were frozen except for the final transformer block (Block 7 of 8), the final layer normalization, and the newly introduced WindowEmbeddings layer, following the fine-tuning recommendations of the base model authors^8^. The fine-tuning dataset and subject partitioning used are described in Section Apnea and Artifacts (AA) Dataset.

**Triplet construction:** Each training step operated on triplets of PPG windows drawn from two morphologically distinct signal categories. For each batch, the anchor and positive sample were drawn from the **same** category, while the negative sample was drawn from the **different** category from the same subject. Since the anchor could belong to either signal category, the model was trained to separate the two morphologies symmetrically in both directions, effectively utilising all available training windows from both categories. For each anchor window, the positive sample was randomly selected from a different window of the same category and subject, while the negative sample was randomly selected from a window of the opposite category from the same subject.

**Training hyperparameters:** The model was optimized using the Adam optimizer^17^ with a learning rate of $3 \times 10^{-4}$, weight decay of $3 \times 10^{-5}$, and momentum parameters $\beta _1 = 0.9$, $\beta _2 = 0.999$, $\epsilon = 10^{-8}$. Training was conducted for 100 epochs with a batch size of 64. No learning rate scheduler was applied. To ensure reproducibility, a fixed random seed of 0 was used throughout, with deterministic algorithm settings enforced. The model checkpoint achieving the lowest validation loss was retained for all subsequent evaluations.

### The signal quality assessment (SQA) methodology

Once the embedding model is sufficiently trained using triplet loss, and meaningful per-window PPG embeddings are attainable; these embeddings can be utilized to detect PPG windows that are significantly different by means of spatial distance in the embedding space. When working with physiological signals that are highly complex such as PPG, it is impractical to establish fixed clustering centroids and expect them to successfully attribute quality assessments throughout a variety of sensor positions and artifact types. Therefore, relative distances are key in this methodology.

Euclidean distances are calculated between each point and a set of reference points that, ideally, represent PPG windows with high signal quality, with the heuristic assumption that, if a point represent a PPG window with significantly lower quality, the median distance between the reference points is substantially less than the median distance between the point and each of the reference points.

In mathematical terms, let $\textbf{r}_1, \textbf{r}_2, \dots , \textbf{r}_N \in \mathbb {R}^d$ be *N* reference embedding vectors. We can define the pairwise Euclidean distance matrix $\textbf{D} \in \mathbb {R}^{N \times N}$, where:

1 $$\begin{aligned} D_{i,j} = \Vert \textbf{r}_i - \textbf{r}_j \Vert _2 \end{aligned}$$

Given matrix $\textbf{D}$, we can extract a set of all non-diagonal upper triangle elements that represent all unique intra-reference Euclidean distances between the pairs as:

2 $$\begin{aligned} \mathcal {S} = \left\{ D_{i,j} \;\Bigg |\; 1 \le i < j \le N \right\} \end{aligned}$$

Let $\textbf{x} \in \mathbb {R}^d$ be the sample point of concern (e.g new PPG window), we can define the set of Euclidean distances between the point $\textbf{x}$ and each of the reference points $\textbf{r}_i \in \mathbb {R}^d, \quad \text {for } i = 1, 2, \dots , N$ as:

3 $$\begin{aligned} \mathcal {P}_x = \left\{ \Vert \textbf{x} - \textbf{r}_i \Vert _2 \;\Bigg |\; i = 1, 2, \dots , N \right\} \end{aligned}$$

Using the sets $\mathcal {S}$ and $\mathcal {P}_x$, we can formulate a heuristic condition using the relative distances as:

4 $$\begin{aligned} \text {Assessment} = {\left\{ \begin{array}{ll} \text {''Lower Quality''}, & \text {if } \dfrac{\operatorname {Median}(\mathcal {P}_x)}{\operatorname {Median}(\mathcal {S})} \ge \textbf{c} \\ \text {''Similar Quality''}, & \text {otherwise} \end{array}\right. } \end{aligned}$$

Where *c* is a fixed threshold set to 2 in this work, deliberately aligned with the triplet loss margin used during fine-tuning, which defines the minimum Euclidean separation the model was trained to enforce between embeddings of morphologically dissimilar windows. A window whose median distance to the reference set exceeds this trained separation boundary is therefore classified as lower quality. Note that *c* was set to 6 for the WCS dataset, as detailed in Section The simulated real-time SQA workflow, to account for the larger embedding distances arising from its cross-device reference configuration.

It is clear that the establishment of a reliable set of reference points is crucial to the success of this methodology. Additionally, it is notable that the set of references allows for a high degree of flexibility that could permit the same model to be utilized in different PPG datasets and conditions. Notably, this distance-based formulation is inherently agnostic to the type of artifact present: any deviation from the morphological characteristics of the reference set, whether it was caused by motion, poor sensor contact, ambient light interference, or physiological factors such as low perfusion, will manifest as increased embedding distance, without requiring prior knowledge of the artifact type or signal degradation mechanism.

### Datasets

Three different datasets were used in order to validate the SQA methodology proposed in this work. The following subsections briefly describe each dataset and present the preprocessing steps taken.

#### Complex system laboratory (CSL) dataset

Finger PPG signals were acquired by a data acquisition system in the Complex Systems Laboratory (CSL) from two pediatric cases in the Pediatric Intensive Care Unit (PICU) at Doernbecher Children’s Hospital, Oregon Health & Science University^9^. The PPG signals were sampled at 125 Hz. Additionally, the dataset included annotations of visual artifacts marked by an independent expert. This dataset can be accessed online at borealisdata.ca.

#### Wearable and clinical devices (WCS) dataset

The WCS dataset provides signals of 18 healthy subjects in two experimental conditions: while sitting stationary and movement (simulated walking in-place)^18,19^. Signals were recorded simultaneously from a clinical-grade device (FlexComp, Thought Technology) providing finger PPG signals at 2048 Hz, and a wrist-worn device (E4, Empatica) providing wrist PPG signals at 64 Hz. The original study was approved by the Independent Review Board of the University of Trento (2017–2019) and conducted in accordance with the principles of the Declaration of Helsinki, and written informed consent was obtained from all participants prior to participation. Only de-identified data were used in the present analysis.

Since this dataset does not include artifact annotations, two assumptions were made, as well as an artifact annotation step was taken before using the dataset for evaluating the SQA methodology. The assumptions were: 1) Finger PPG recordings acquired while stationary via the FlexComp device are generally clean and contain PPG pulses with ideal morphology, and 2) Wrist PPG recordings collected while performing the simulated walking are generally noisy and corrupted.

**Fig. 7:**
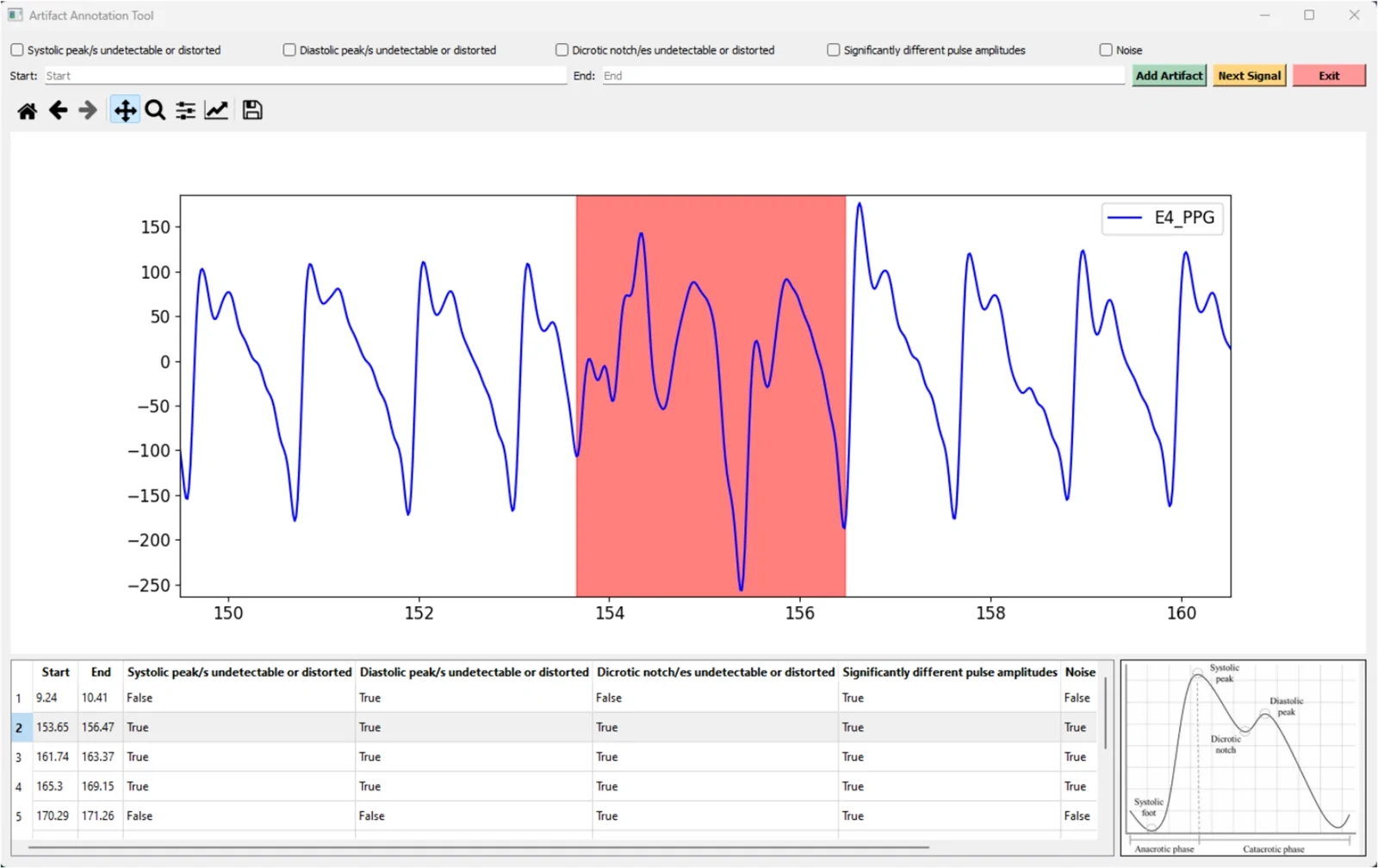
WCS Artifact annotator app with the necessary information and functionality for a reliable and consistent annotation.

When it came to the stationary recordings collected via the E4 device on the wrist, artifacts were annotated by an independent expert using a custom-designed annotator app for this purpose, the layout of which, along with an example annotated signal, is shown in Fig. 7. The annotator app allowed the annotator to classify each artifact duration as one or more of 5 different classifications:

1. Systolic peak/s undetectable or distorted
2. Diastolic peak/s undetectable or distorted
3. Dicrotic notch/es undetectable or distorted
4. Significantly different pulse amplitudes
5. Noise (implying that all the above classes are true, due to somewhat stochastic noise)

Furthermore, to ensure that assumption 1 holds true for most of the stationary recording durations, the areas annotated as ‘Noise’ artifacts in the wrist PPG signals were eliminated from the respective finger PPG recordings.

#### Apnea and artifacts (AA) dataset

This dataset provided neck PPG signals (at 75 Hz) from healthy subjects while exhibiting different respiratory states: shallow breathing, slow breathing, and breath-holding apnea events, as well as additional external and intrinsic artifacts such as talking, swallowing, coughing, yawning, sensor rubbing, body movements, and head movements (right/left, rotation and up/down)^11,20^. Each recording of 140 seconds contained three periods of the simulated event, with 20-second rest periods in between where subjects were spontaneously breathing. Events’ onsets and offsets were marked during the data acquisition process, and each recording was normalized thereafter. In this work, PPG signals collected from 12 subjects who performed respiratory rate modulation as well as artifact events were used in two stages; for fine-tuning and later for SQA evaluation, based on the type of recording. The original study was approved by the Local Ethics Committee of Imperial College London (ICREC ref.: 18IC4358). The study was conducted in accordance with the principles of the Declaration of Helsinki, and written informed consent was obtained from all participants. Only de-identified data were used in this analysis.

Fine-tuning the base model required the introduction of significantly different PPG segments to form the set of triplet data points. To fulfill this task, PPG signals collected during the simulated apnea and sensor rubbing were used; as these two event types produce signals with substantially different qualities and morphologically distinct characteristics, making them well-suited for learning an embedding separation via triplet loss. Subjects were partitioned into training (9 subjects, randomly sampled) and validation (remaining subjects) sets. The two fine-tuning categories were defined as: windows fully overlapping with annotated apnea events, and windows fully overlapping with annotated sensor-rubbing events. These recordings were subsequently excluded from the SQA evaluation, leaving the recordings corresponding to: body movement, head right/left, head rotation, head up/down, coughing, yawning, swallowing and talking, of which the corresponding annotated events were considered artifacts.

### Signal processing, embeddings visualization, and the real-time SQA workflow

This subsection outlines the key implementation details that support the proposed SQA methodology. First, the preprocessing steps are explained, which were applied to the raw PPG signals to segment the data and put it in shape for the PPG window embedding model. Second, a visualization of the learned embedding space is presented, using the CSL dataset, which serves to illustrate how the model internally is able to partition the PPG segments based on the learned representations. Finally, the simulated real-time SQA pipeline is detailed, emulating how the model can be deployed in streaming settings using fixed-length sliding windows and dynamic reference-based scoring.

#### Signal processing

**Fig. 8:**
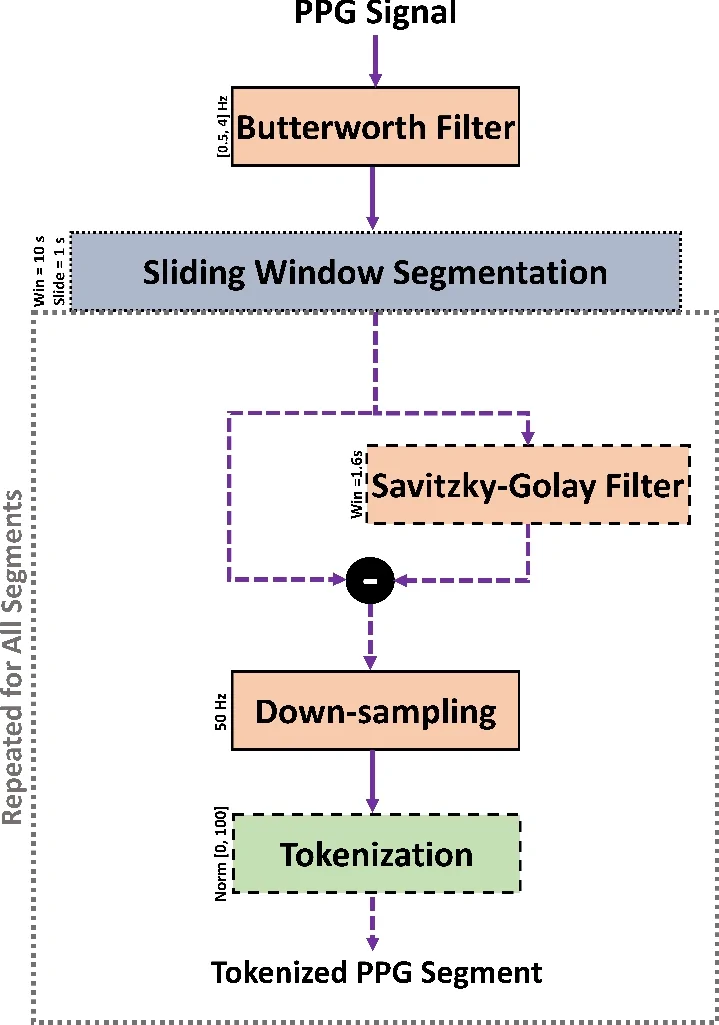
PPG signal preprocessing pipeline.

The same PPG preprocessing steps were applied to all three datasets, consisting of a ${1}^{\textrm{st}}$-order [0.5 - 4] Hz bandpass Butterworth filter, followed by a 10-s sliding window segmentation scheme with a slide of 1 second in order to simulate a real-time processing window of sampled and bandpass-filtered PPG. For each PPG segment, a ${2}^{\textrm{nd}}$ order Savitzky–Golay filter with a window size of 1.6 seconds was applied to a copy of the PPG segment, which was then subtracted from it to further reduce low-frequency baseline deviations, as done in previous published work^21^. Thereafter, the PPG segment is downsampled to 50 Hz and tokenized by scaling to the range [0, 100], complying with the original PPG-PT paper^8^. These preprocessing steps are visualized in Fig. 8.

#### Visual validation of learned embedding representations - WCS dataset

In order to qualitatively assess the structure of the learned embedding space, the model’s output representations for the WCS dataset were visualized using Uniform Manifold Approximation and Projection (UMAP). UMAP is a nonlinear dimensionality reduction technique that preserves both local and global structure, making it particularly well-suited for exploring the distribution of high-dimensional data. In this context, UMAP serves as a tool to examine whether the learned embeddings reflect a tangible separation between ‘clean’ and ‘artifact’ PPG segments. Observing a partitioning behaviour in the projected space supports the validity of the reference-guided distance-based SQA assessment methodology.

**Fig. 9:**
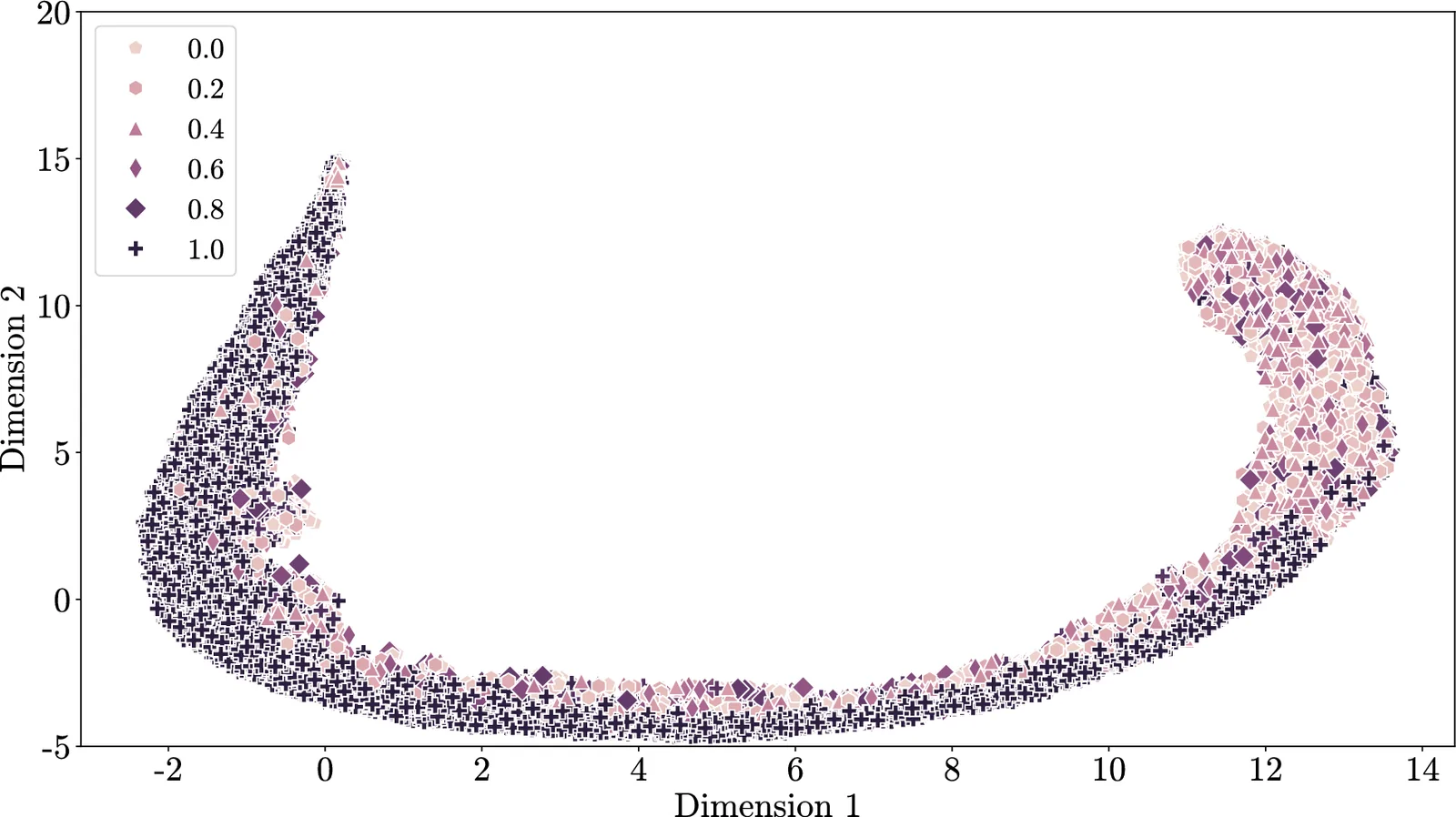
UMAP of the embedding space corresponding to the **WCS** dataset. Each point represents a PPG segment, color- and marker-coded by artifact proportion: light-colored pentagon markers indicate segments with no annotated artifacts (proportion = 0.0), progressing through intermediate shapes and colors, as shown in the legend, to dark cross markers indicating fully corrupted segments (proportion = 1.0). The degree of spatial separation between the two reflects the model’s ability to distinguish clean from artifact-corrupted segments in the learned embedding space.

Figure 9 shows the UMAP visualization of the WCS dataset, where each point is color- and marker-coded based on the proportion of artifacts in the respective PPG segment, from 0 (no annotated artifacts in the segment) to 1 (the entire segment was annotated as artifact).

The UMAP shows a clear attempt by the embedding model to push PPG segments that are suffering from high levels of artifact contamination far away from segments that are less contaminated, and ideally, the furthest away from clean PPG segments. Certainly, a middle region where some points are confused is expected, most likely due to subject-specific variability, as well as the manual artifact annotation accuracy, and in addition, could be due to the embedding model performance.

The supplementary Figures S3 and S4 show the UMAP visuals for the CSL and AA datasets, respectively, illustrating similar overall separation, although understated due to the reduced number of PPG segments with an artifact proportion greater than 0.8, especially in the CSL dataset.

These observations support the general idea and the rationale behind the proposed SQA technique, which heavily relies on the formation of a suitable reference set.

#### The simulated real-time SQA workflow

Establishing the set of reference points was done in two different ways, depending on the dataset, allowing for a thorough evaluation of the SQA methodology. When evaluating the methodology on the CSL and AA datasets, the first 10 windows were used to generate the reference set, due to the notable absence of significant artifacts at the beginning of the recordings, which can translate to requiring the user to remain still for about 15 seconds at the beginning of the monitoring session in a real-life setting.

In case of the WCS evaluation, the reference set was gathered from finger PPG signals acquired during the stationary recordings, and those references were used to detect artifacts in the wrist PPG signals during the annotated stationary recordings and the movement recordings. This was done to resemble using a pre-acquired set of references, in both a generalized and personalized fashion. The number of reference data points varied depending on the examined evaluation scheme.

**Personalized reference set:** The reference set was sampled from the stationary finger PPG signal of the same subject. For instance, when evaluating the SQA methodology on subject A’s wrist PPG signals, the reference set would be randomly sampled from subject A’s finger PPG signal of the stationary recording. In this scheme, the number of sampled windows generating the reference set was set to 10, 15, and 20.

**Generalized reference set:** In this case, a subset of subjects was randomly sampled and used to generate the reference pool of finger PPG signals, from which a reference set was formed by randomly sampling a fixed number of windows from each subject, resulting in a populated reference set that can be used for artifact detection on the remaining subjects. A subset of 40%, 50%, 60% and 70% subjects was examined, and the number of sampled windows from each subject was set to 2, 5 and 10.

When considering the CSL and AA datasets, the ground truth was set to 1 if at least 10% of the window was annotated as an artifact, and 0 otherwise. In case of evaluating on the WCS dataset; the ground truth was set to 1 if at least 10% of the window was annotated as class 4 or class 5 (’Significantly different pulse amplitudes’ or ’Noise’) in the stationary recordings, or if the window belonged to the movement recordings, and it was set to 0 otherwise. Furthermore, $\textbf{c}$ (operating on equation 4) was set to 2 for datasets CSL and AA, while it was set to 6 for dataset WCS due to the different choice of reference pools and the relatively larger distance anticipated.

To characterise the computational requirements of the proposed framework, the inference time of the fine-tuned model was measured on a standard CPU over 100 repeated forward passes using a single tokenized 10-second window as input. The mean inference time was *45.85 ± 1.69* ms (range: 42.28–49.93 ms), well within the 1-second slide interval of the real-time pipeline, yielding an effective output latency of 1 s with approximately 954 ms of headroom per cycle. The computational cost per inference is 198.85 MFLOPs. The fine-tuned model comprises 447,769 parameters in total (60,697 fine-tuned, corresponding to the WindowEmbeddings head and the final transformer block), with a memory footprint of 1.71 MB in single-precision floating point, making it suitable for deployment on resource-constrained wearable platforms. For reference, the base PPG-PT model prior to the addition of the WindowEmbeddings head comprises 443,943 parameters as reported in^8^.

### Evaluation metrics

The well-known classification evaluation metrics are used in this work: sensitivity, precision, F1-score, and accuracy. In addition to the Cohen’s kappa measure. These metrics are aggregated over all subjects in the respective dataset and summarized by the mean ± standard deviation. When the term average or mean is stated, it refers to the measure averaged over the positive and negative classes for each subject, before it is aggregated over all the subjects.

Cohen’s kappa (*κ*) is a statistical measure of inter-rater agreement for categorical outputs. This measure takes into account the possibility of agreement occurring due to chance. It is commonly used to evaluate the performance of a classifier against the ground truth, when the output is categorical. The Cohen’s Kappa measure is essentially defined as: $\kappa = \frac{p_o - p_e}{1 - p_e}$, where $p_o$ is the observed agreement, represented by the proportion of instances where the predicted and true labels agree. $p_e$ is the probability that the two labelers agree by chance, given the distribution of the labels. It can also be calculated from a 2x2 confusion matrix by means of the true positive (TP), false positive (FP), true negative (TN), and false negative (FN) proportions as:

$$\begin{aligned} \kappa = \frac{2 \times (TP \times TN - FN \times FP)}{(TP + FP) \times (FP + TN) + (TP + FN) \times (FN + TN)} \end{aligned}$$

The value *κ* is bound between −1 and 1, and can be interpreted as shown in Table 9 below:

**Table 9:** Interpretation of Cohen’s kappa *κ* coefficient^22^.

*κ* **Value**	Strength of Agreement
*< 0*	Poor (less than chance)
*0.00 - 0.20*	Slight
*0.21 - 0.40*	Fair
*0.41 - 0.60*	Moderate
*0.61 - 0.80*	Substantial
*0.81 - 1.00*	Almost perfect

## Conclusion

This work introduced a methodology for real-time signal quality assessment (SQA) of photoplethysmography (PPG) signals. The approach is based on fine-tuning a pre-trained decoder-only transformer model (PPG-PT) using triplet loss to learn embeddings that reflect morphological differences between PPG windows. These embeddings are used to detect low-quality segments by computing their relative distances from a set of reference embeddings assumed to represent high-quality signals, without requiring handcrafted features or signal-specific heuristics.

The methodology was evaluated on three publicly available datasets: CSL (providing finger PPG), AA (providing neck PPG), and WCS (providing wrist PPG as well as finger PPG), encompassing a variety of artifact types and acquisition conditions. On the CSL dataset, the method achieved strong agreement with expert annotations, with an overall accuracy of $93.66 \pm 0.38\%$, F1-score of $89.98 \pm 0.39\%$, and Cohen’s kappa of *0.80 ± 0.01*. Results on the AA dataset showed more moderate performance (overall accuracy: $71.96 \pm 8.25\%$, F1-score: $69.92 \pm 8.04\%$, $\kappa = 0.42 \pm 0.15$), likely due to the increased inter-subject variability and the nature of artifacts encountered in neck-worn PPG signals.

On the WCS dataset, both personalized and generalized reference configurations were tested. The generalized approach, in particular, demonstrated consistently high performance when using reference windows from 50% or more of the subjects (F1-score *≈* 95%, $\kappa \approx 0.90$), outperforming the personalized approach and highlighting its potential for population-level deployment.

While the results indicate that the proposed methodology can generalize across sensor locations and artifact types, several limitations remain. The performance is sensitive to the representativeness and quality of the reference set, and will degrade when the distribution of the evaluation data differs significantly from the references, as seen in a subset of subjects in the personalized WCS evaluation. In addition, the method currently assumes that a reliable set of reference segments can be defined or anticipated *a priori*, which may not always be feasible in uncontrolled or real-world settings.

Furthermore, the decision threshold *c* is currently fixed and dataset-dependent; future work could explore adaptive selection based on the statistical properties of the intra-reference distance set $\mathcal {S}$.

From a deployment perspective, the method is computationally lightweight once embeddings are generated and can operate in a real-time framework, as characterized in detail in Section The simulated real-time SQA workflow, which points at its suitability for deployment on resource-constrained wearable platforms, enabling potential integration in wearable systems for continuous monitoring.

Future work could explore unsupervised or semi-supervised methods for selecting reference embeddings automatically, improve robustness to inter-device variability, and investigate the extension to other physiological signals and multimodal input sources. Additionally, the sensitivity of the proposed methodology to the choice of window length has not been formally evaluated; future work could systematically assess performance across different window lengths to identify optimal configurations for different sensor locations and artifact types.

## Supplementary Information

Supplementary Information.

## Data Availability

The Complex System Laboratory (CSL) Dataset is publicly available at: https://borealisdata.ca/dataset.xhtml?persistentId=doi:10.5683/SP2/SJAKCB. The Wearable and Clinical Devices (WCS) Dataset and the Apnea and Artifacts (AA) Dataset were obtained from the original authors and are not publicly available; however, they are available from the original authors of the respective publications upon reasonable request. Data generated during the current study are available from the corresponding author upon reasonable request.

## References

[1] Castaneda, D., Esparza, A., Ghamari, M., Soltanpur, C. & Nazeran, H. A review on wearable photoplethysmography sensors and their potential future applications in health care. *Int. J. Biosens. Bioelectron.* **4**(4), 195 (2018). 10.15406/ijbsbe.2018.04.00125 PMC6426305 30906922

[2] Charlton, P. H. et al. Wearable Photoplethysmography for Cardiovascular Monitoring. *Proc. IEEE* **110**, 355–381, 3 (2022). 10.1109/JPROC.2022.3149785 PMC7612541 35356509

[3] Charlton, P. H. et al. The 2023 wearable photoplethysmography roadmap. *Physiol. Meas.* **44**, 111001, 11 (2023). 10.1088/1361-6579/acead2 PMC10686289 37494945

[4] Elgendi, M., Norton, I., Brearley, M., Abbott, D. & Schuurmans, D. Detection of a and b waves in the acceleration photoplethysmogram. *BioMed. Eng. Online* **13**, 1–18, 9 (2014). 10.1186/1475-925X-13-139 PMC4190349 25252971

[5] Moscato, S., Giudice, S. L., Massaro, G. & Chiari, L. Wrist Photoplethysmography Signal Quality Assessment for Reliable Heart Rate Estimate and Morphological Analysis. *Sensors* **22**, 5831, 8 (2022). 10.3390/s22155831 PMC9370973 35957395

[6] Kim, J., Cho, H., Lee, M. & Kim, S. B. Multi-source partial domain adaptation with gaussian-based dual-level weighting for ppg-based heart rate estimation. *Knowl.-Based Syst.* **309**, 112769 (2025).

[7] Pillai, A., Spathis, D., Kawsar, F. & Malekzadeh, M. Papagei: Open foundation models for optical physiological signals. *International Conference on Learning Representations* **2025**, 48230–48261 (2025).

[8] Davies, H. J., Monsen, J. & Mandic, D. P. Interpretable Pre-Trained Transformers for Heart Time-Series Data Vol. 7, arXiv preprint: arXiv:2407.20775 (2024).

[9] W. Karlen, J. M. Ansermino, and G. Dumont, Adaptive pulse segmentation and artifact detection in photoplethysmography for mobile applications, * Proceedings of the Annual International Conference of the IEEE Engineering in Medicine and Biology Society, EMBS*, pp. 3131–3134, (2012). 10.1109/EMBC.2012.6346628 23366589

[10] Almeida, A. et al. Design of a Photoplethysmography Signal Qualifier: A Geometric Approach for Mobile Applications. In *Available at SSRN 4942633* (2024).

[11] García-López, I., Pramono, R. X. A. & Rodriguez-Villegas, E. Artifacts classification and apnea events detection in neck photoplethysmography signals. *Med. Biol. Eng. Comput.* **60**, 3539–3554, 12 (2022). 10.1007/s11517-022-02666-1 PMC9646626 36245021

[12] Jun, W., Hui, H., Handong, Y., Pengfei, X. & Zhong, J. A lightgbm-based method for the signal quality assessment of wrist photoplethysmography. *Phys. Eng. Sci. Med.* (4), 10.1007/s13246-025-01616-z (2025). 40830716

[13] Karlen, W., Raman, S., Ansermino, J. M. & Dumont, G. A. Multiparameter respiratory rate estimation from the photoplethysmogram. *IEEE Trans. Biomed. Eng.* **60**(7), 1946–1953 (2013). 10.1109/TBME.2013.2246160 23399950

[14] Pimentel, M. A. F. et al. Toward a robust estimation of respiratory rate from pulse oximeters. *IEEE Trans. Biomed. Eng.* 10.1109/TBME.2016.2613124 (2017). PMC6051482 27875128

[15] Kachuee, M., Kiani, M. M., Mohammadzade, H. & Shabany, M. Cuff-less high-accuracy calibration-free blood pressure estimation using pulse transit time, In *2015 IEEE international symposium on circuits and systems (ISCAS)*, pp. 1006–1009, (IEEE, 2015).

[16] Balntas, V., Riba, E., Ponsa, D. & Mikolajczyk, K. Learning local feature descriptors with triplets and shallow convolutional neural networks. *Procedings of the British Machine Vision Conference* **2016**, 1–119 (2016).

[17] Kingma, D. P. & Ba, J. Adam: A method for stochastic optimization. arXiv preprint: arXiv:1412.6980 (2014).

[18] Bizzego, A., Gabrieli, G. & Esposito, G. Deep Neural Networks and Transfer Learning on a Multivariate Physiological Signal Dataset. *Bioengineering* **8**(3), 35 (2021). 10.3390/bioengineering8030035 PMC8058952 33800842

[19] Bizzego, A., Gabrieli, G., Furlanello, C. & Esposito, G. Comparison of Wearable and Clinical Devices for Acquisition of Peripheral Nervous System Signals. *Sensors* **20**(11), 6778 (2020). 10.3390/s20236778 PMC7730565 33260880

[20] Garcia-Lopez, I. & Rodriguez-Villegas, E. Characterization of Artifact Signals in Neck Photoplethysmography. *IEEE Trans. Biomed. Eng.* **67**(10), 2849–2861 (2020). 10.1109/TBME.2020.2972378 32142413

[21] Abdulsadig, R. S. & Rodriguez-Villegas, E. A novel computational signal processing framework towards multimodal vital signs extraction using neck-worn wearable devices. *Sci. Rep.* **14**22368. (2024). 10.1038/s41598-024-72184-7 PMC11437265 39333140

[22] Landis, J. R. & Koch, G. G. The Measurement of Observer Agreement for Categorical Data. *Biometrics* **33**, 159, 3 (1977). 843571

