# Supplementary material for: Domain-independent PPG signal quality assessment framework via representation learning
Source: Sci Rep. 2026 Jul 20;16:33073. doi: 10.1038/s41598-026-58783-6 (PMC13683049; doi:10.1038/s41598-026-58783-6)
Supplement: Supplementary file 1 [file 41598_2026_58783_MOESM1_ESM.pdf]

Supplementary Figures

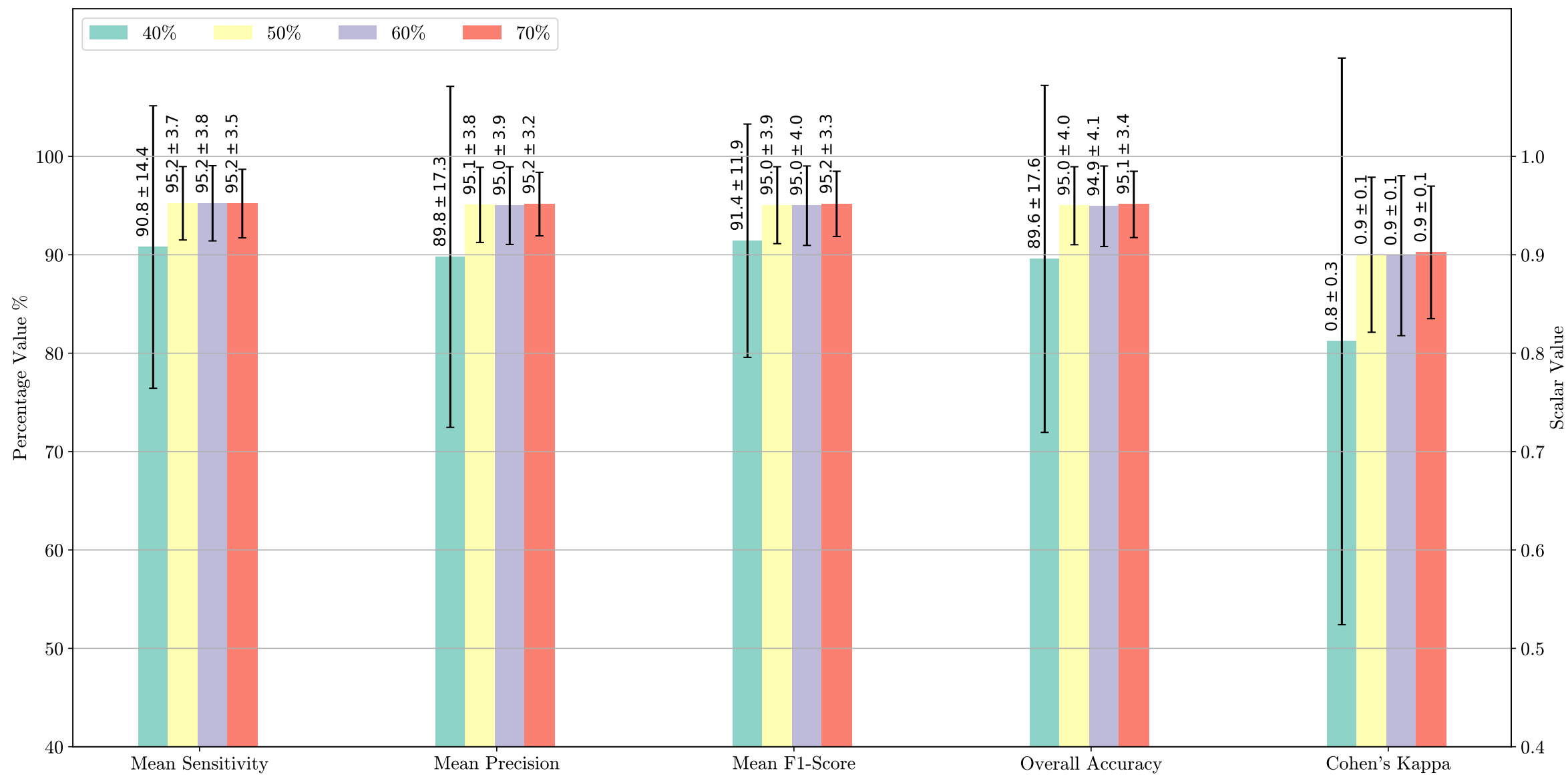

Figure S1: Barplots of the different evaluation metrics (mean sensitivity, mean precision, mean F1-score, overall accuracy, and Cohen's kappa) when **2** reference windows were sampled from 40%, 50%, 60%, and 70% of the subjects.

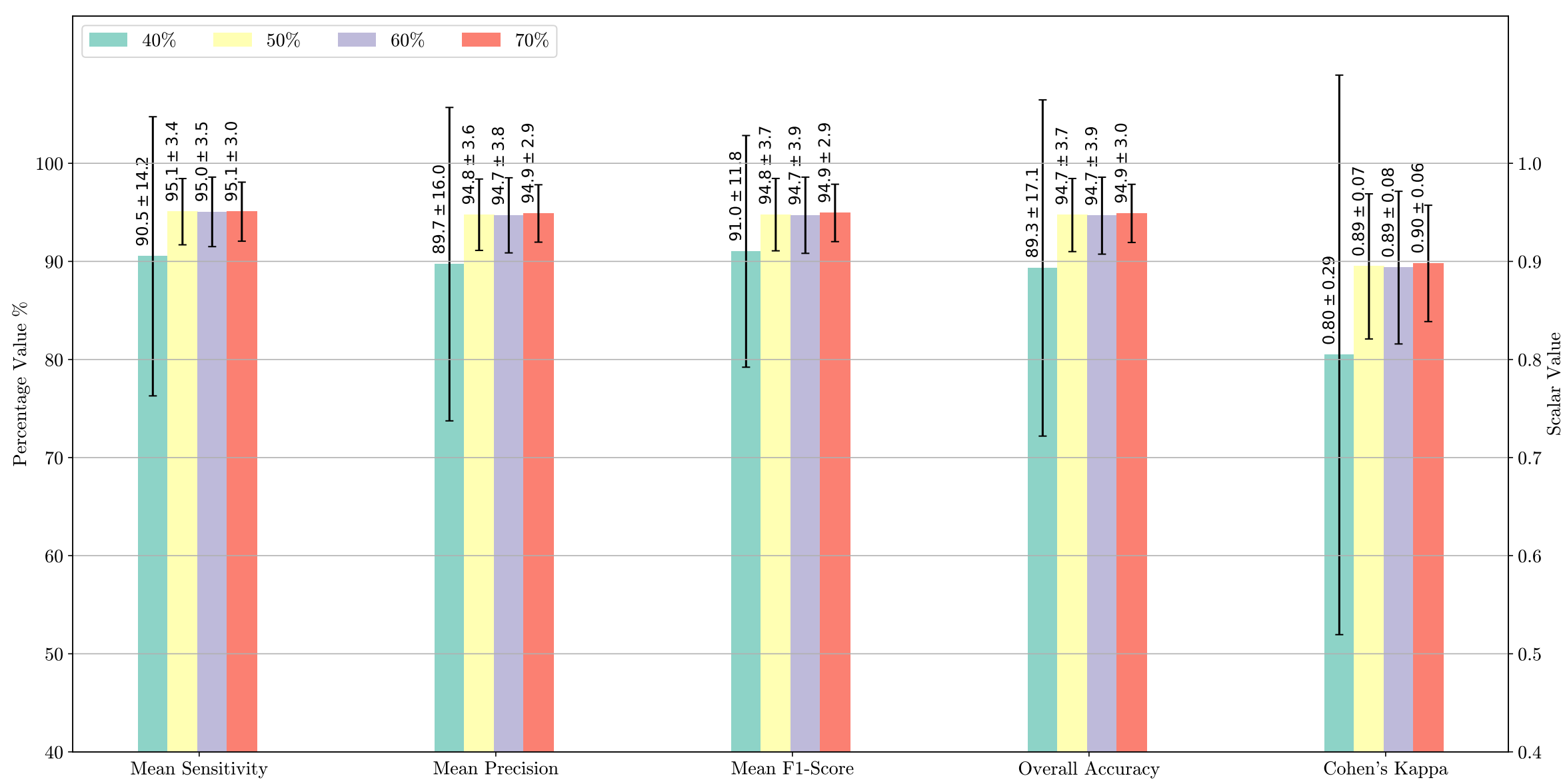

Figure S2: Barplots of the different evaluation metrics (mean sensitivity, mean precision, mean F1-score, overall accuracy, and Cohen's kappa) when **10** reference windows were sampled from 40%, 50%, 60%, and 70% of the subjects.

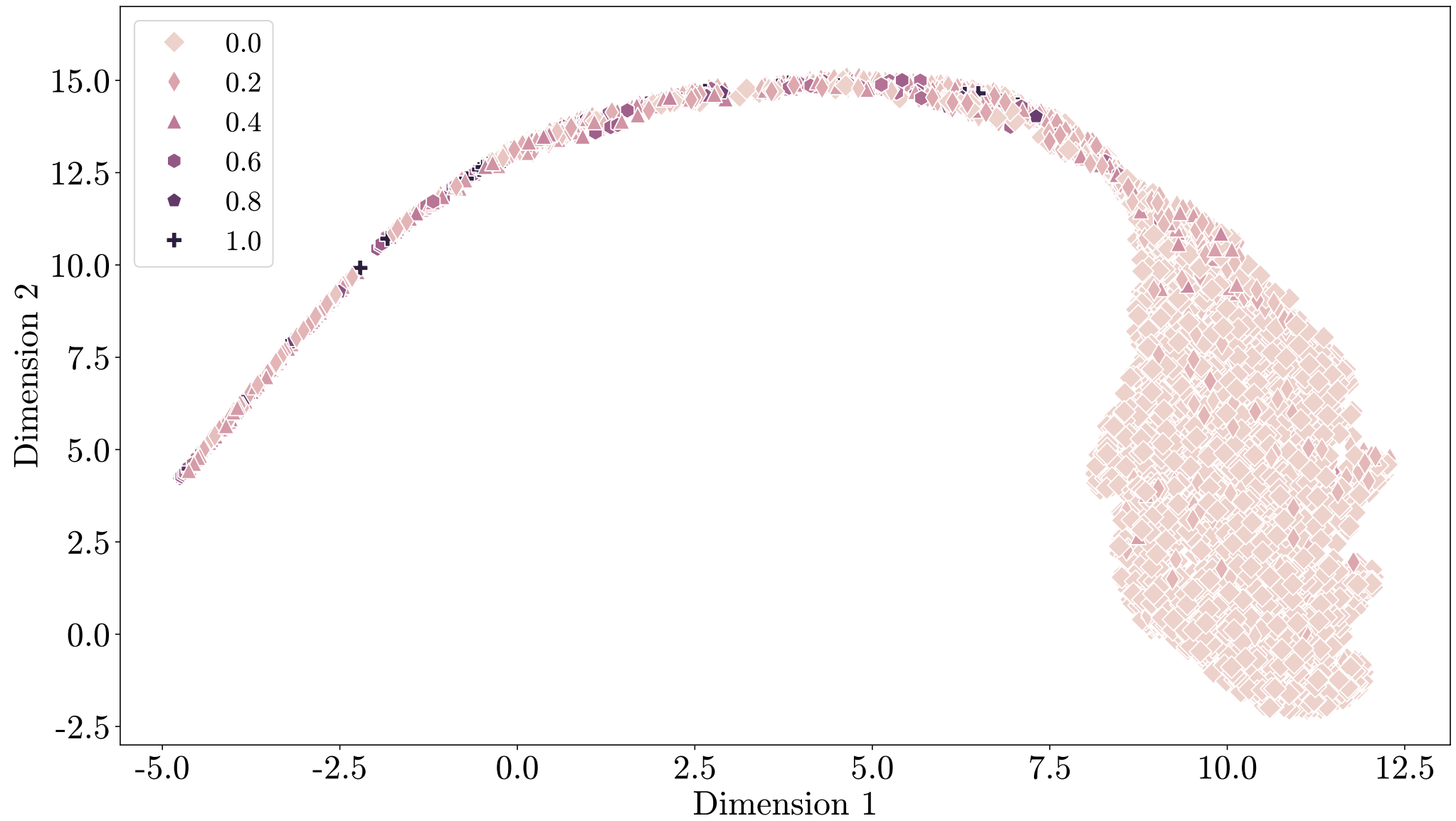

Figure S3: UMAP of the embedding space corresponding to the **CSL** dataset, showing the different landmark proportions of annotated artifacts within the respective PPG segments in unique colors and shapes. Light-colored pentagon markers indicate segments with no annotated artifacts, progressing through intermediate shapes and colors to dark cross markers indicating fully corrupted segments. The separation between markers of differing artifact proportions illustrates the model's capacity to partition PPG segments by morphological quality in the learned embedding space.

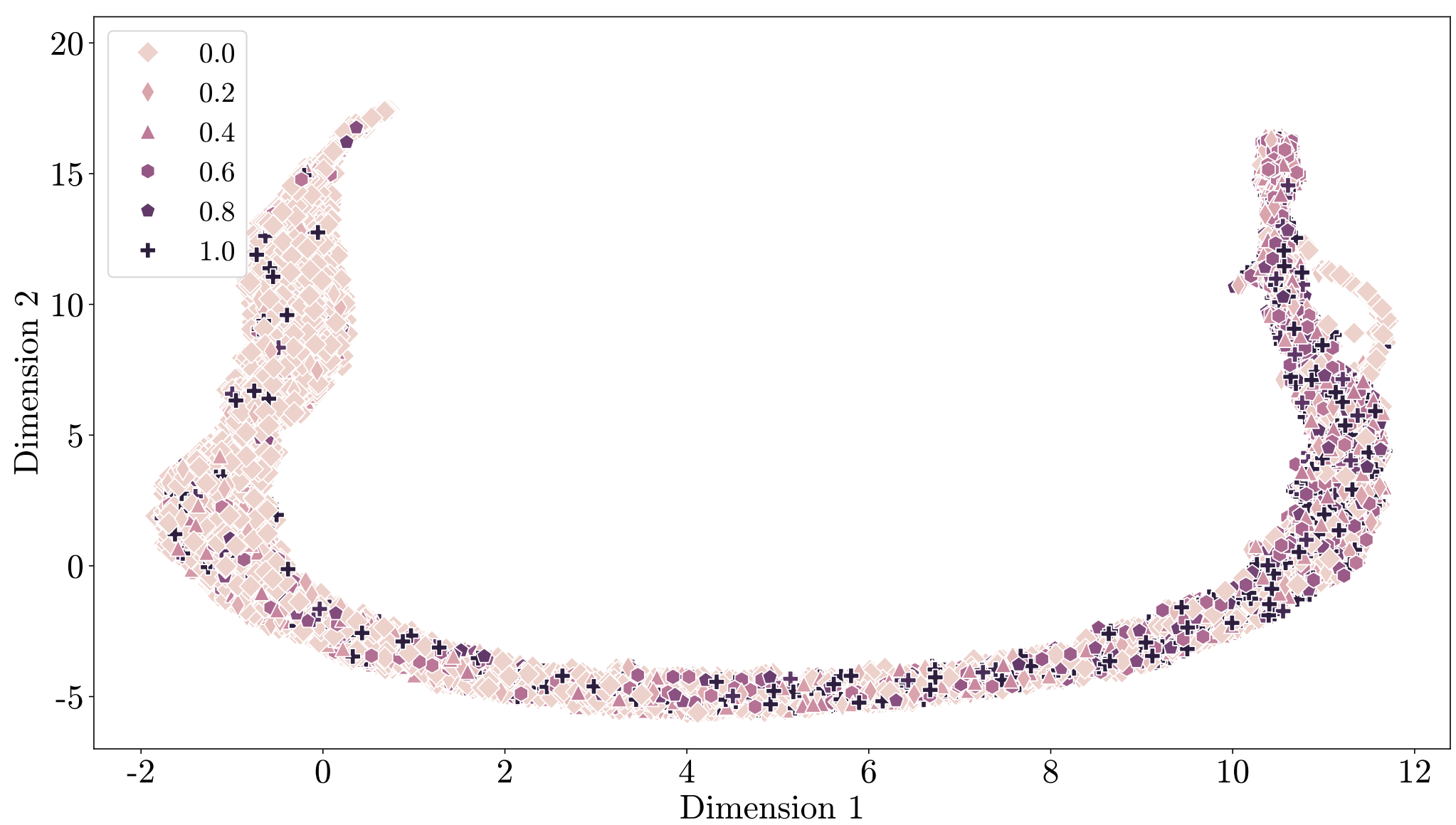

Figure S4: UMAP of the embedding space corresponding to the **AA** dataset, showing the different landmark proportions of annotated artifacts within the respective PPG segments in unique colors and shapes. Light-colored pentagon markers indicate segments with no annotated artifacts, progressing through intermediate shapes and colors to dark cross markers indicating fully corrupted segments. The separation between markers of differing artifact proportions illustrates the model's capacity to partition PPG segments by morphological quality in the learned embedding space.
